# Digital single-operator cholangioscopy to guide selective cannulation of complex biliary strictures

**DOI:** 10.1007/s00464-022-09665-1

**Published:** 2022-10-19

**Authors:** Janine B. Kastelijn, Paul Didden, Auke Bogte, Leon M. G. Moons, Frank P. Vleggaar

**Affiliations:** Department of Gastroenterology and Hepatology, University Medical Center Utrecht, P.O. Box 85500, 3508 GA Utrecht, The Netherlands

**Keywords:** Biliary drainage, Biliary strictures, Digital single-operator cholangioscopy, Endoscopic retrograde cholangiopancreaticography, Selective cannulation

## Abstract

**Background:**

Selective cannulation and stenting of complex, tight, and/or angulated biliary strictures under endoscopic retrograde cholangiopancreaticography (ERCP) can be challenging. Digital single-operator cholangioscopy (SOC) may facilitate guidewire advancement through the stricture with endoscopic visual guidance. We aimed to describe a case series on clinical outcomes of this technique for selective cannulation, when used after failed conventional ERCP attempts.

**Methods:**

Consecutive patients who underwent therapeutic digital SOC for selective cannulation of biliary strictures after failed conventional ERCP were retrospectively included.

**Results:**

Ten patients with a malignant (*n* = 6) or benign (*n* = 4) biliary stricture were included. Digital SOC-assisted selective guidewire insertion and stent placement across the biliary stricture were technically successful in five (50%) patients. Bilirubin levels improved in all patients with technical success. One (10%) patient developed a post-ERCP pancreatitis.

**Conclusions:**

Technically successful cannulation of biliary strictures with digital SOC was achieved in half of patients in whom cannulation with conventional ERCP failed, sparing them more invasive interventions. Stricture opacification during the failed ERCP was seen in all technically successful SOCs.

**Supplementary Information:**

The online version contains supplementary material available at 10.1007/s00464-022-09665-1.

Biliary strictures are caused by malignant (e.g., cholangiocarcinoma) or benign (e.g., post-liver transplantation or in primary sclerosing cholangitis [PSC]) conditions. Endoscopic retrograde cholangiopancreaticography (ERCP) is the preferred technique for managing these strictures and achieving biliary drainage; however, guidewire placement across complex or high grade strictures can be challenging [1]. In anastomotic strictures after liver transplantation, for example, reported overall success rate of ERCP as primary treatment was 57%, ranging from 40 to 92% [2]. The main reason for endoscopic failure is failed guidewire insertion [1]. When ERCP fails, more invasive procedures such as endoscopic ultrasound-guided or percutaneous transhepatic biliary drainage are required.

Digital single-operator cholangioscopy (SOC) is mostly known for its benefit in treating complex biliary stones and diagnosing biliary strictures. Complementary to ERCP, SOC-assisted guidewire placement might also be helpful for cannulating complex strictures. It allows the guidewire to be advanced through the biliary stricture under direct endoscopic visual guidance [3, 4]. In addition, it allows non-contrast-guided access of intrahepatic ducts, which may decrease the risk of post-ERCP cholangitis [5]. Data on technical feasibility and clinical outcomes of digital SOC-assisted selective cannulation after failed conventional ERCP attempts, however, are limited.

Therefore, we aimed to describe the clinical outcomes of this rescue technique in a case series of 10 patients and to provide an overview of available literature.

## Patients and methods

### Patients

Consecutive patients who underwent digital SOC (SpyGlass™ DS System, Boston Scientific, USA) after its introduction at our institution in June 2017 up until September 2021 were screened for eligibility. We retrospectively included all patients who underwent therapeutic digital SOC for selective cannulation of complex biliary strictures after failed conventional ERCP. Patients with biliary strictures in whom SOC was performed for diagnostic reasons were excluded. All ERCPs and digital SOCs were performed by experienced endoscopists. In case of multiple procedures, the first digital SOC-procedure of each patient was registered. This study was approved by our Institutional Medical Research Ethics Committee (MREC-number 20/664).

### Digital SOC-assisted guidewire placement technique

All procedures were performed under deep sedation with propofol. First, the biliary stricture was assessed using cholangiography after contrast injection. Digital SOC was performed only when attempts to advance an 0.035-inch guidewire (straight or angled tip) with a standard ERCP-cannula were unsuccessful. After inserting the cholangioscope into the bile duct, the stricture was visualized endoscopically. A long guidewire (Jagwire, 0.035 inch, 450 cm, straight tip, Boston Scientific) was passed through the working channel and directed through the stricture under direct visualization. Guidewire passage proximal of the stricture could be shown with fluoroscopy. Finally, a plastic or metal stent was introduced over the guidewire, if necessary preceded by balloon dilation.

### Outcomes and definitions

Main objective was technical success, defined as successful guidewire insertion and stent placement across the targeted biliary stricture. Secondary outcomes were improvement of bilirubin level, reintervention, and adverse event (AE) rate. Post-procedural bilirubin level was defined as the lowest bilirubin value measured within 60 days after digital SOC and before an additional biliary intervention was performed. Reintervention was defined as any additional endoscopic or percutaneous biliary intervention after the initial digital SOC. AEs occurring within 30 days were defined and graded according to the ASGE lexicon [6]. The presence of contrast passage through the stricture during preceding ERCP was registered. Patients were followed until death or last contact. Descriptive statistical analysis was performed with STATA version 15.1; Fig. 2 was designed with R version 3.5.1.

## Results

In total, 126 digital SOC-procedures were performed in 104 patients for various indications. Of these, ten patients underwent therapeutic digital SOC for selective cannulation of biliary strictures. Mean age was 68 (± 13.9) years and six (60%) patients were male. Stricture etiology was malignant in 6 (60%) and benign in 4 (40%) patients. The majority (7/10) of strictures were located in the liver hilum or intrahepatically. Biliary opacification upstream of the stricture was achieved cholangiographically in five patients prior to SOC. Causes of failure of the preceding ERCP attempt were a complete stop without any contrast filling of the intended bile duct system (*n* = 5), or because guidewires could not pass the stenosis (*n* = 5). Baseline characteristics are shown in Table 1. Data of each individual patient are shown in Appendix 1.

**Table 1:** Baseline characteristics

Age in years, mean (± SD)	68 (± 13.9)
Male sex, *n* (%)	6 (60)
WHO PS, median (IQR)	1 (1–1)
Radiographical imaging modality of biliary stricture, *n* (%)^a^	
MRCP MRI	8 (80)
CT-liver	1 (10)
Abdominal US	1 (10)
Etiology, *n* (%)	
Malignant^b^	6 (60)
Benign^c^	4 (40)
Bilirubin level before drainage in μmol/L, median (IQR)	106 (79–131)
Location stricture, *n* (%)	
Common hepatic duct	3
Common hepatic duct toward intrahepatic	2
Left hepatic duct	3
Right hepatic duct	1
Right hepatic duct toward posterior	1

*CT* computed tomography, *IQR* interquartile range, *MRCP* magnetic resonance cholangiopancreatography, *SD* standard deviation, *US* ultrasound, *WHO PS* world health organization performance status

^a^Biliary strictures were confirmed with MRCP. If unavailable, CT or abdominal US were used

^b^Cholangiocarcinoma (*n* = 6)

^c^Primary Sclerosing Cholangitis (*n* = 2), cholecystectomy clips (*n* = 1), radiation-induced fibrosis (*n* = 1)

**Fig. 1:**
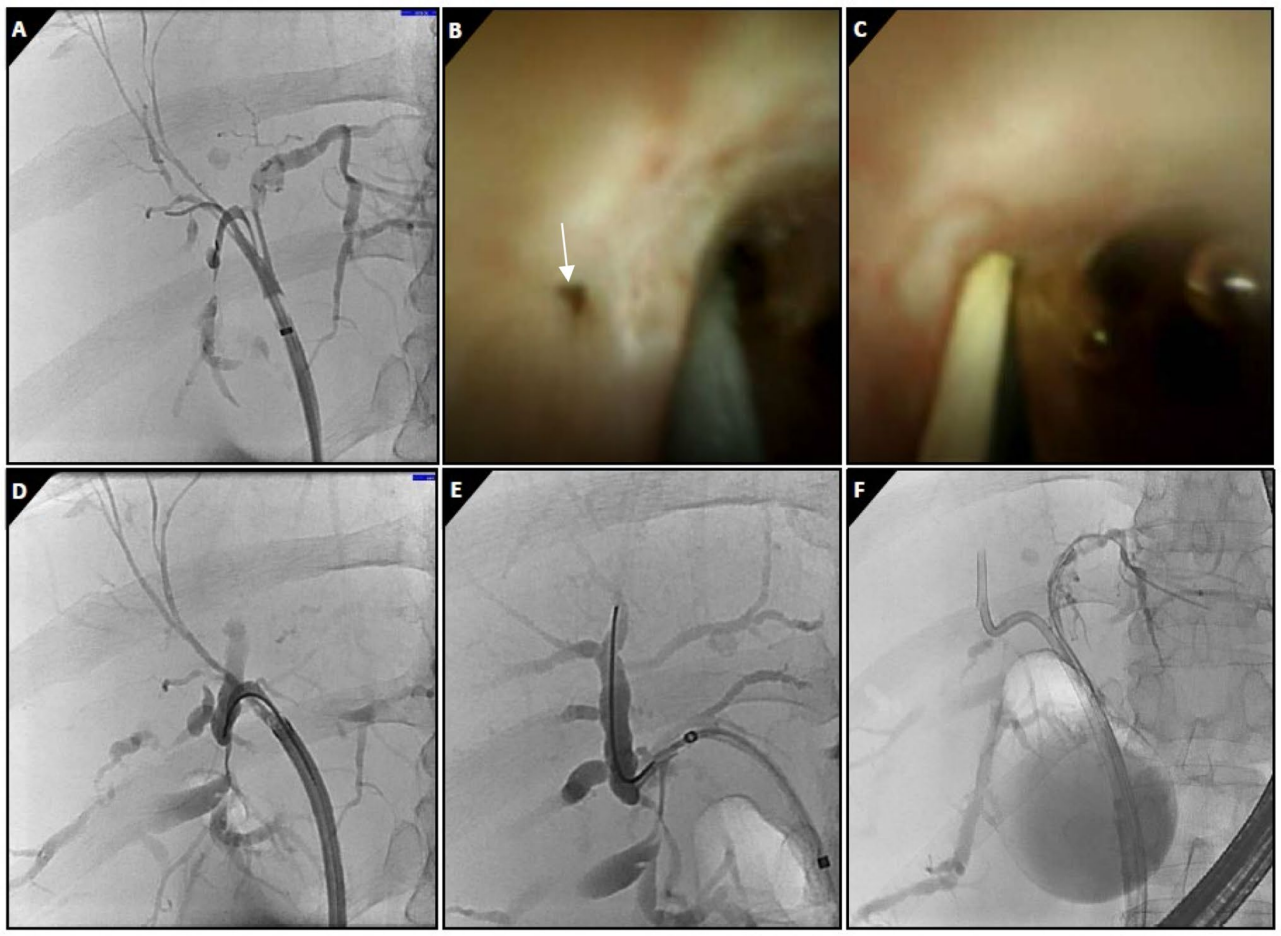
60-years-old female with intrahepatic cholangiocarcinoma, located centrally in the liver compressing the hilar bile ducts, resulting in obstructive jaundice. **A** Occlusion cholangiogram shows a tight stricture of the right posterior bile duct. However, a guidewire is preferentially being advanced into the non-dilated right anterior bile duct. **B** Cholangioscopic visualization of the small orifice of the right posterior bile duct (arrow). **C** SOC-assisted guidewire placement using fluoroscopy (**D**). **E** Deep cannulation and contrast injection of a dilated segmental bile duct. **F** Insertion of a 18 cm 7Fr plastic endoprothesis after balloon dilation of the stricture

**Fig. 2:**
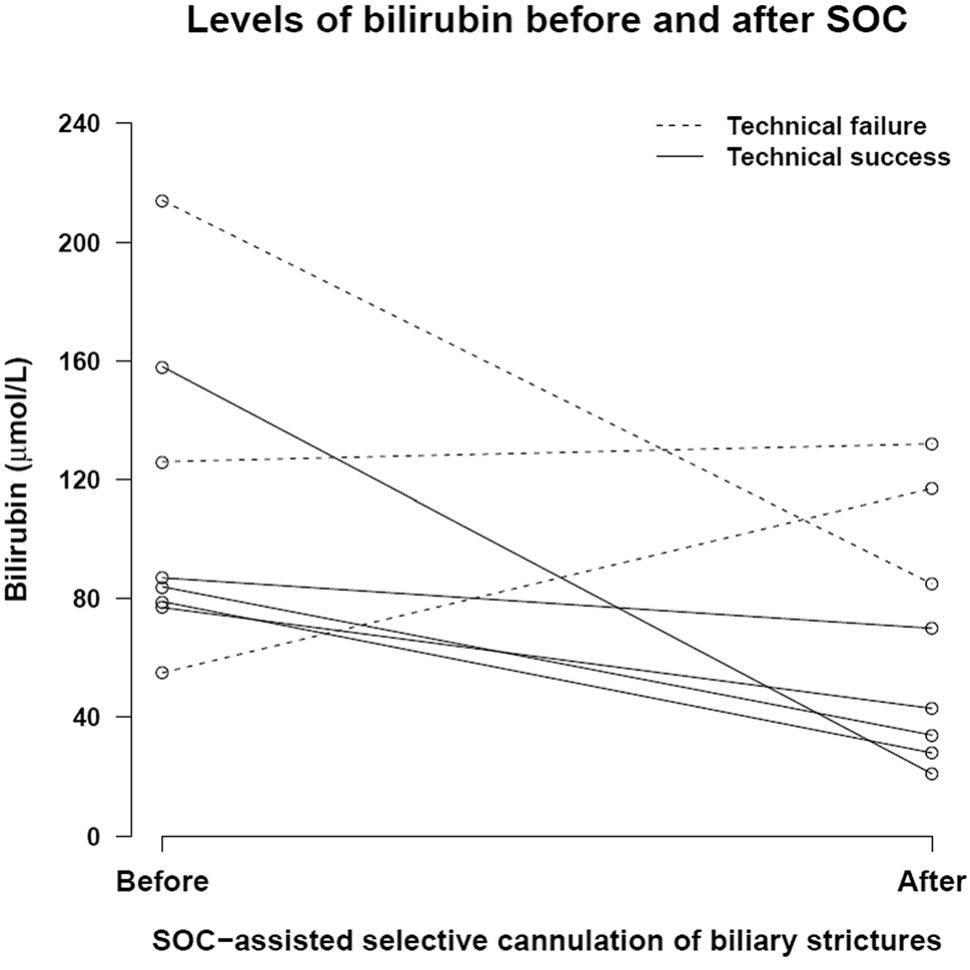
Levels of bilirubin before and after SOC, by technical success. Two patients were omitted, in whom post-procedural bilirubin (after technical failure) was missing

Digital SOC-assisted selective guidewire insertion and stent placement across the biliary stricture were technically successful in five (50%) patients: two with benign (radiotherapy-induced and post-cholecystectomy) and three with malignant (cholangiocarcinoma) etiology (Fig. 1, video 1). Aimed guidewire insertion was not achieved because it was impossible to identify the (correct) ostium (*n* = 3), the ostium could not be cannulated with the guidewire (*n* = 1), or because the Spyglass could not pass the narrowed distal common bile duct in a PSC-patient. Three strictures were related to cholangiocarcinoma and two to PSC. Contrast passage through the stricture with upstream opacification during preceding ERCP was seen in all technically successful SOCs and in none of the failed SOCs. Bilirubin levels improved in all five patients with successful guidewire insertion and stent placement, from a median level of 84 (IQR 79–87) umol/L to 34 (IQR 28–43) umol/L. (Fig. 2).

Nine (90%) patients required one or more repeat biliary reinterventions. Four of these were treated with a temporary internal–external PTCD after digital SOC had been technically unsuccessful. Five patients underwent one or more repeated ERCPs for various indications, including elective stent exchange or progressive stenting, and cholangitis due to stent dysfunction more than 30 days after SOC. Only one AE was reported within 30 days after ERCP with SOC-assisted guidewire insertion. This patient developed a fatal post-ERCP pancreatitis after successfully draining a malignant subhilar stricture. Six (60%) patients died before time of analysis (January 2022), with a median survival of 348 (IQR 149–492) days (Table 2).

**Table 2:** Outcomes

Technical success, *n* (%)	5 (50)
Bilirubin level (μmol/L) after successful SOC, median (IQR)	34 (28–43)
Adverse events < 30 days, *n* (%)	1 (10)
Biliary reintervention, *n* (%)	9 (90)
Survival in days, median (IQR)	348 (149–492)
Duration of follow-up in days, median (IQR)	652 (295–1137)

Data of each individual patient are shown in Appendix 1

## Discussion

This case series of 10 patients demonstrated that introducing digital SOC-assisted guidewire placement as a rescue modality after failed ERCP results in successful selective cannulation and drainage of complex biliary strictures in half of patients, sparing these patients more invasive interventions. Bilirubin levels improved in all these patients. Contrast passage upstream of the stricture during preceding cholangiography was seen in all patients with technically successful SOC and in none of the failed procedures.

Technical success rates of SOC-assisted selective guidewire placements have been reported in small series and varied between 60 and 100% [7–13]. A review of literature was performed and an overview of available studies reporting ≥ 5 patients is provided (Appendix 2). In the largest series to date, technical success rate of SOC-assisted guidewire placement after failed conventional approach was 70% (21/30 procedures) [7]. Higher technical success rates were achieved in benign biliary strictures compared with malignant strictures (88% vs. 46%, respectively, *p* = 0.02) [7]. Benefit was seen especially in post-liver transplant strictures. We did not find different success rates between benign and malignant strictures, possibly due to the small sample size and relatively high proportion of PSC-patients. Orifice identification and cannulation of irregular, extensive, and inflammatory strictures, as seen in cholangiocarcinoma but also in PSC, can be more challenging compared with regular and more short-segment benign strictures, such as liver transplantation-associated strictures. It suggests that not only benign or malignant etiology, but also morphology of the biliary stricture and surrounding mucosa may affect the ability to visualize the stricture orifice and hence success rates of cannulation. Importantly, we found that all successful SOC-assisted guidewire placements were preceded by contrast passage of the stricture during cholangiography. This is a new finding which has not been reported in previous studies. Cholangiographic stricture localization guides the direction in which the cholangioscope should be advanced and helps identifying the stricture with SOC. Searching an unopacified intrahepatic duct is much more challenging, especially when bile duct narrowing or malignant infiltration already starts below the hilum, as frequently seen in PSC or Bismuth ≥ 3 perihilar cholangiocarcinoma. Our study suggests that in these situations, SOC may not be beneficial in achieving selective biliary drainage. Future studies should be performed to confirm if the absence of contrast passage during the failed conventional ERCP attempt is a risk factor associated with failure of SOC indeed and could be used as selection criterium for subsequent SOC-assisted attempts.

The safety profile of selective digital SOC-assisted guidewire insertion is based on few small series. In our series, one patient died of post-ERCP pancreatitis. Previously reported procedure-related AE rates, consisting of cholangitis, pancreatitis, and bleeding, varied between 0% (of 5 and 15 patients)[10, 13] and 16.7% (of 23 patients) [7]. Endoscopic treatment of complex malignant strictures is associated with a relatively high risk of post-ERCP cholangitis, because adequate drainage of such strictures and opacified ducts is difficult to achieve [14]. SOC allows non-contrast-guided access of intrahepatic ducts, which may decrease the risk of post-ERCP cholangitis [5].

Several strategies can be applied to optimize visibility and chances of successful SOC-assisted selective cannulation. Careful pre-drainage mapping of the biliary tree with MRCP to determine the exact location and extent of the obstruction will help to guide selective cannulation. Flushing with sufficient normal saline to improve image quality for direct endoscopic inspection of the ostium is crucial. Furthermore, the cholangioscope can be used to inject contrast solution very closely to the targeted stricture, which may help visualize the lumen within and proximally of the biliary stricture. It is also necessary to use a long (> 4.5 m) guidewire to be able to remove the SOC before stent placement, as the therapeutic armamentarium that can be advanced through the working channel of the cholangioscope is still limited.

This study has limitations. First, patients were retrospectively identified and data were retrospectively collected from patient records. In addition, the small size of our cohort did not allow statistical analysis.

In conclusion, this case series of 10 patients showed that digital SOC-assisted guidewire insertion and stent placement was achieved in half of the biliary strictures where cannulation with conventional ERCP had failed. Opacification of the biliary stricture during the failed ERCP was seen in all technically successful SOCs.

## Supplementary Information

Below is the link to the electronic supplementary material.

Supplementary file1 (DOCX 38 KB)

Supplementary file2 (M4A 677 KB) Video 1 60-years-old female with intrahepatic cholangiocarcinoma, located centrally in the liver compressing the hilar bile ducts, resulting in obstructive jaundice. There is a tight stricture of the right posterior bile duct. However, a guidewire is preferentially being advanced into the non-dilated right anterior bile duct. The small orifice of the right posterior bile duct is visualized cholangioscopically. SOC-assisted guidewire placement is performed, followed by deep cannulation, balloon dilation of the stricture and insertion of an 18 cm 7Fr plastic endoprothesis.
