# Supplementary material for: Digital single-operator cholangioscopy to guide selective cannulation of complex biliary strictures
Source: Surg Endosc. 2022 Oct 19;36(12):9476–80. doi: 10.1007/s00464-022-09665-1 (PMC9652227; doi:10.1007/s00464-022-09665-1)
Supplement: Supplementary file 1 — Supplementary file1 (DOCX 38 KB) [file 464_2022_9665_MOESM1_ESM.docx]

Appendix 1. Individual patient data of digital SOC-assisted selective cannulation of biliary strictures

| Case | Age | Sex | WHO PS | Etiology | Bilirubin before^a^ | Stricture location, course, and estimated length^b^ | Contrast passage^c^ | TS^d^ | Bilirubin  after ^a^ | AEs <30 days | Repeat intervention |
| --- | --- | --- | --- | --- | --- | --- | --- | --- | --- | --- | --- |
| 1 | 35 | F | 0 | Benign: PSC | 55 | CHD, tortuous, 10 mm | No | No | 117 | No | 1 ERCP (new attempt after failed SOC) |
| 2 | 64 | M | 1 | Benign:  radiotherapy induced | 158 | LHD, straight 12 mm | Yes | Yes | 21 | No | 4 elective ERCPs (once with SOC) for stent change and progressive stenting |
| 3 | 73 | F | 1 | Benign: cholecystectomy clips | 84 | CHD, straight, 22 mm | Yes | Yes | 34 | No | 2 ERCPs (1 suspected cholangitis; 1 stent dislocation and change to uSEMS) |
| 4 | 80 | M | 1 | Benign: PSC | 126 | RHD, tortuous, 25 mm | No | No | 132 | No | Internal-external PTCD |
| 5^e^ | 60 | F | 1 | Malignant: CCA | 87 | RHD segmental towards right posterior, tortuous,  15 mm | Yes | Yes | 70 | No | 2 ERCPs (1 stent dislocation with duodenal perforation; 1 cholangiosepsis stent change) |
| 6 | 63 | M | 1 | Malignant: CCA | 130 | CHD towards left, straight,  5 mm | No | No | - | No | 2 internal-external PTCD |
| 7 | 79 | M | 1 | Malignant: CCA | 79 | CHD, straight,  5 mm | Yes | Yes | 28 | PEP (fatal) | No |
| 8 | 83 | F | 0 | Malignant: CCA | 214 | LHD, straight,  40 mm | No | No | 85 | No | 3 internal-external PTCD |
| 9 | 74 | M | 1 | Malignant: CCA | 77 | LHD, tortuous 18 mm | Yes | Yes | 43 | No | 3 ERCPs (2x cholangitis with stent change; 1x change PS to uSEMS) |
| 10 | 66 | M | 1 | Malignant: CCA | 131 | CHD towards intrahepatic, straight,  18 mm | No | No | - | No | 1 internal-external PTCD |
| Abbreviations: AEs = Adverse events; CCA = cholangiocarcinoma; CHD = common hepatic duct; ERCP = Endoscopic retrograde cholangiopancreaticography; F = female; LHD = left hepatic duct; M = male; mm = millimetre; PEP = post-ERCP pancreatitis; PS = Plastic stent; PSC = Primary sclerosing cholangitis; PTCD = Percutaneous transhepatic cholangial drainage; RHD = right hepatic duct; TS = technical success; uSEMS = uncovered Self-expandable metal stent; WHO PS = World Health Organization Performance Status.  Footnotes: ^a^ Bilirubin is measured in μmol/L; ^b^ Stricture length was estimated radiographically and/or cholangiographicaaly; ^c^ Contrast passage during conventional ERCP, prior to introducing Spyglass; ^d^ Five patients did not achieve technical success due to the following reasons: it was impossible to identify the (correct) ostium (case 6, 8 and 10), the ostium could not be cannulated with the guidewire (case 4), or because the Spyglass could not pass the narrowed distal common bile duct (case 1); ^e^ illustrated in figure and video 1 | | | | | | | | | | | |

Appendix 2. Literature overview of studies on SOC-assisted selective cannulation of biliary strictures

| **Author,  year of publication** | **Country** | **Patients treated with SOC-assisted selective cannulation,  n (% of total included patients)** | **Study design** | **Treatment years** | **Mean age in years** | **Male sex,  n (%)** | **Biliary stricture etiology** | **Successful selective cannulation, n (%)** | **Adverse events, n (%)** | **Bilirubin level im-provement after technical success,**  **n (%)** |
| --- | --- | --- | --- | --- | --- | --- | --- | --- | --- | --- |
| Woo et al, 2016^13^ | Korea | 15 (100) | Case series | 2010-2014 | 60 | 13 (87) | Post-LDLT | 9 (60) | None | 9 (100%) |
| Hüsing-Kabar et al, 2017^8^ | Germany | 16 (62) | Prospective observational study | 2016 | 52 | 9 (56) | Post-LT^1^ | 16 (100) | NR^2^ | NR |
| Ogura et al, 2017^11^ | Japan | 7 (13) | Prospective study | 2016 | NR^2^ | NR^2^ | NR^2^ | 5 (71) | NR^2^ | NR |
| Martins et al, 2017^10^ | Brazil | 5 (100) | Case series | NR | NR | NR | Post-LT | 5 (100) | None | NR |
| Lenze et al, 2018^9^ | Germany | 10 (15) | Retrospective cohort study | 2015-2017 | NR^2^ | NR^2^ | NR^2^ | 8 (80) | NR^2^ | NR |
| Bokemeyer et al, 2019^7^ | Germany | 23 (100) | Retrospective cohort study | 2015-2018 | 51 (median) | 10 (44) | Benign:  12 (52)^3^ Malignant:  11 (48)^4^ | 18 (78) | 5 (17)^5^ | NR |
| Weigand et al, 2021^12^ | Germany | 31 (17)^6^ | Retrospective cohort study | 2016-2017 | 64 | 66 (56) | NR^2^ | 27 (87)^6^ | NR^2^ | NR |
| **Abbreviations:** LDLT=Living donor liver transplantation; LT=Liver transplantation; NR=Not reported  **Footnotes:** ^1^ of which 1/26 has had LDLT; ^2^ Outcome not reported for the selective cannulation subgroup separately; ^3^ Liver transplantation-associated (n=9), Primary sclerosing cholangitis (n=2), Secondary sclerosing cholangitis (n=1); ^4^ Cholangiocarcinoma (n=7), pancreascarcinoma (n=1), other malignancies (n=3); ^5^ reported per examination: 30 digital SOCs were performed in 23 patients. Consisting of pancreatitis (n=2), cholangitis (n=2) and bleeding requiring endoscopic treatment (n=1). ^6^ reported per examination: 180 digital SOCs were performed in 117 patients, of which 31 selective guidewire insertions. | | | | | | | | | | |

**Appendix 2:** Three case series (of 5, 16 and 15 patients) with post-liver transplant biliary strictures reported successful guidewire insertion in 100%^8, 10^ and 60%^13^. The latter included only living (and no deceased) donor liver transplantations, in which more complex bile duct anatomy with more angulated and peripheral anastomoses might explain the lower success rate.^1^ Three studies reported patients who underwent selective cannulation as part of larger cohorts of patients who were treated with digital SOC for various other indications. These studies reported success rates between 71%, 80%, and 87% (in 7, 10, and 31 patients, respectively).^9, 11, 12^ Outcomes were not reported by stricture aetiology (benign or malignant) in these studies.
